# Evaluation of the sexual function of men in Kazakhstan during 2021–2022: A cross‐sectional study

**DOI:** 10.1002/hsr2.1142

**Published:** 2023-02-28

**Authors:** Marat Sikhymbaev, Dinara Ospanova, Andrey Grzhibovsky, Merkhat Akkaliyev, Turar Kurmanbekov, Shynar Tanabayeva, Timur Saliev, Sagat Altynbekov, Ildar Fakhradiyev

**Affiliations:** ^1^ Department of Medicine S.D. Asfendiyarov Kazakh National Medical University Almaty Republic of Kazakhstan; ^2^ Department of Medicine Al‐Farabi Kazakh National l University Almaty Republic of Kazakhstan; ^3^ Department of Medicine Northern State Medical University Arkhangelsk Russian Federation; ^4^ Department of Medicine Semey Medical University Almaty Republic of Kazakhstan

**Keywords:** aging, erectile dysfunction, population‐based research, sexual dysfunction, sexual satisfaction

## Abstract

**Background and Aims:**

Assessing male sexual function is an important public health issue in every country. In Kazakhstan, there are currently no reliable statistics on male sexual function. The study aimed at the assessment of sexual function in men in Kazakhstan.

**Methods:**

Men between the ages of 18 and 69 from Astana, Almaty, and Shymkent, three of Kazakhstan's biggest cities, were included in the cross‐sectional study in 2021–2022. A standardized and modified Brief Sexual Function Inventory (BSFI) tool was used for participants' interviews. The World Health Organization STEPS questionnaire was employed to gather sociodemographic information, including smoking and alcohol use.

**Results:**

Respondents from three cities: *n* = 283 from Almaty, *n* = 254 from Astana, and *n* = 232 from Shymkent were interviewed. All participants' average age was 39.2 ± 13.4. Kazakhs made up 79.5% of the respondents by nationality; 19.1% who answered questions on physical activity verified that they were involved in high‐intensity labor. According to the BSFI questionnaire, the respondents from Shymkent had an average total score of 2.82 ± 0.92, (*p* ≤ 0.05), which was higher than the total scores of respondents from Almaty (2.69 ± 0.87) and Astana (2.69 ± 0.95). A relationship was found between sexual dysfunction and age indicators over 55 years. Participants with overweight had a relationship with sexual dysfunction with an odds ratio (OR): 1.84 (*p* = 0.01). According to the smoking factor, in study participants with sexual dysfunction, a relationship was also determined, OR: 1.42; 95% confidence interval (CI): 0.79–1.97 (*p* = 0.001). The presence of high‐intensity activity (OR: 1.58; 95% CI: 0.04–1.91), and physical inactivity (OR: 1.49; 95% CI: 0.89–1.97) were associated with the presence of sexual dysfunction, *p* ≤ 0.05.

**Conclusions:**

Our research indicates that men over 50 who smoke, are overweight, and are physically inactive are at risk for sexual dysfunction. Early health promotion may be the most effective method to reduce the negative effects of sexual dysfunction on the health and wellbeing of men over 50.

## 1 INTRODUCTION

One of the most important aspects of life is sexual function, which has a significant impact on the body, mind, social behavior, and quality of life.^1^ In fact, problems with sexual function may lead to psychosocial issues, lowering work productivity, and a decrease in quality of life.

Most of the published reports in this area have been focused on the evaluation of specific diagnostic and therapeutic strategies.^2^ However, wide population‐based studies have not been conducted in developing countries, particularly in Central Asia and Kazakhstan. The need for large‐scale studies of sexual function in various Asian populations may help determine not only risk factors but also the effectiveness of the treatment of male sexual dysfunction.^3^

The use of a short questionnaire to assess sexual function in men has proven useful in some studies.^4^, ^5^ The Male Sexual Function Inventory (Brief Sexual Function Inventory [BSFI]) was published by O'Leary et al.^6^ The questionnaire is a concise, self‐administered, and clinically useful tool for this kind of epidemiological research.^7^ Sexual dysfunctions are a group of disorders that are typically characterized by clinically significant disturbances in response to sexual pleasure.^8^ Thus, the BSFI tool and overall BSFI score can be useful for finding strong correlations between functional areas and comprehensive analysis.^9^

Depending on factors such as culture, race, and health, different countries have varying incidence rates for sexual issues.^10^ Local health institutions must pay an attention to the sociocultural landscape of their region as well as the incidence and epidemiology of sexual dysfunctions. It might help to reduce the risk of harmful sexual behavior and improve life quality.

According to a survey of the literature, Kazakhstan has not yet seen the completion of such studies. This study aims to evaluate men's sexual behavior in the Republic of Kazakhstan using three major cities as examples (Almaty, Astana, and Shymkent).

## 2 METHODS

### 2.1 Study subjects

The study was conducted in the period 2021–2022. This cross‐sectional study included men aged 18–69 from three major cities in Kazakhstan (Astana, Almaty, and Shymkent). Participation in the study is completely voluntary.

The fundamental goal of the sample design is to ensure that the indicators measured represent the state of the nation as a whole and that the sample is national in scope and coverage. Using a multistage sampling technique, a list of every polyclinic medical facility in a given city was compiled as part of the sampling procedure. The sample included men who visited a polyclinic at their place of residence as part of a preventive examination. Systematic random sampling was used to recruit no more than 30 people per day from each polyclinic. It was separated into 10 age categories, including 18–24, 25–29, 30–34, 35–39, 40–44, 45–49, 50–54, 55–59, 60–64, and 65 and older, using a weighted, multistage, cluster sampling methodology. In each age category, a stratification was also done by sex (men).

The study sample size was determined using the special World Health Organization (WHO) STEPS tool. The following indicators were employed for the analysis: probability value for 95% confidence interval (CI): 1.96; the estimated prevalence of risk factors: 0.5; error: 0.05; designer effect: 1.5; expected response rate: 70%. As a result of the calculation, the sample size *n* = 769 was obtained.

### 2.2 Inclusion criteria

Men population only aged >18 were included (age from 18 to 69); residents of three following cities: Astana, Almaty, and Shymkent; agreement for participation.

### 2.3 Exclusion criteria

Population permanently residing in the following premises and locations: boarding schools (orphanages), specialized institutions for minors in need of social assistance and rehabilitation, social service institutions, hospitals, and other healthcare organizations, the barracks; buildings owned or transferred to the use of religious organizations, prisons, correctional institutions or medical and labor dispensaries, and persons without a permanent residence.

### 2.4 Data collection

Participants were interviewed using the standardized and adapted BSFI tool.^11^ In addition, we collected sociodemographic data (including smoking and alcohol) using the STEPS WHO questionnaire.^12^

The BFSI is a qualified sexual functioning self‐report meter. The indicator covers sexual desire and satisfaction, erectile function, and ejaculatory function, as well as an assessment of the problems of desire, erection, and ejaculation. The BSFI is a self‐administered 11‐item questionnaire in which the first 10 items cover functional aspects of male sexuality such as sexual desire (2 items), erection (3 items), ejaculation (2 items), and 3 points focus on how problematic these aspects are for patients. The last point covers all kinds of sexual satisfaction.^2^

Respondents were asked to report their experiences in the last 30 days. Because the BFSI validates the spectrum of sexual activity, it does not generate any points. Most likely, each domain is considered separately.^13^ The element's scaling ranges from 0 (no feature, big problem, etc.) to 4 (good feature, no problem, etc.); the overall score is calculated by summing the item's scores. Low scores mean poorer function.^14^

STEPS is a WHO standardized but flexible system for monitoring major noncommunicable disease risk factors for countries through questionnaires and physical and biochemical measurements. It is coordinated and supervised by the national authorities of the implementing country. STEPS surveys are typically household‐based and conducted by interviewers using scientifically selected samples.^15^

The BSFI questionnaire was translated by two reproductive health experts fluent in English, Russian, and Kazakh into two languages (Russian and Kazakh) (Appendixes A1 and B1).

The BSFI and STEPS WHO questionnaires were uploaded to the “HealthTrack” mobile application (https://apps.apple.com/kz/app/health-track/id1589077331) for further use by the interviewers. All interviewers who conducted the survey were GCP certified.

### 2.5 Statistical analysis

Statistical analysis was conducted using SPSS software (version 25.0; IBM SPSS Inc.). The results are presented as *M* ± SD. Qualitative characteristics were described in absolute (*n*) and relative values (%). BSFI internal consistency was assessed using Cronbach's *α* test. Multivariate logistic regression was performed for the analysis of risk factors for sexual dysfunction (overall satisfaction rate ≥2x points), and *p* < 0.05 was considered statistically significant for all analyses. Analysis of variance was chosen for the statistical test, as the samples from the groups were independent. Tukey's honestly significant difference for the unequal test was used to test for pairwise differences because there was a difference in the number of patients in each group. Univariate analysis was adjusted for age and gender. The *χ* ^2^ test was used to establish the statistical significance of differences between groups.

## 3 RESULTS

In this study, a total of *n* = 769 respondents were interviewed, of which *n* = 283 (36.8%) were from Almaty city, *n* = 254 (33.0%) from Astana, and *n* = 232 (30.2%) from Shymkent.

Depending on the language of the survey, *n* = 513 (66.7%) participants preferred to use the questionnaire in Russian, while *n* = 256 (33.3%) respondents used the questionnaire in Kazakh.

The mean age of all *n* = 769 participants was 39.2 ± 13.4 years. The average body mass index (BMI) was 26.5 ± 4.8 (min–max = 13.8−80.6).

The general characteristics of the participants included in the study (*n* = 769) are presented in Table 1. Among all participants, respondents in the age group of 18–24 years and 35–39 years were *n* = 113 (14.7%) and *n* = 112 (14.6%), respectively. The number of participants in the age category 3 was equal to *n* = 110 (14.3%). Among the respondents, the smallest share was made up of respondents of the age group of 60–64 years and 65 years and older, in *n* = 38 (4.9%) and *n* = 34 (4.4%) cases.

**Table 1:** General characteristics of the participants included in the study (*n* = 769).

Characteristics	*n*, %
Age categories/(*M*, SD)	
18–24	113 (14.7)/20.8 ± 2.1
25–29	102 (13.3)/27.3 ± 1.34
30–34	110 (14.3)/31.9 ± 1.4
35–39	112 (14.6)/37.1 ± 1.4
40–44	68 (8.8)/41.8 ± 1.4
45–49	72 (9.4)/46.9 ± 1.3
50–54	68 (8.8)/52 ± 1.5
55–59	52 (6.8)/56.9 ± 1.4
60–64	38 (4.9)/61.9 ± 1.4
65 and older	34 (4.4)/66.7 ± 1.5
Body mass index/(*M*, SD)	
<25	256 (33.3)/22.0 ± 1.8
25–29.9	345 (44.9)/26.7 ± 1.3
>30	168 (21.9)/33.6 ± 4.8
Education level	
No school education	5 (0.7)
Completed elementary (4kl)	1 (0.1)
Finished secondary (9kl)	33 (4.3)
Finished secondary (11kl)	187 (24.3)
Higher	339 (44.1)
Postgraduate	180 (23.4)
Refuses to answer	24 (3.2)
Nationality	
Kazakh	611 (79.5)
Russian	78 (10.1)
Uzbeks	21 (2.7)
Ukrainians	8 (1.0)
Uighurs	19 (2.5)
Tatars	6 (0.8)
Other	26 (3.4)
Refuses to answer	
Marital status	
Single	194 (25.2)
Married	536 (69.7)
Married but separated	6 (0.8)
Divorced	24 (3.1)
Widower	7 (0.9)
Is in a civil marriage	2 (0.3)
Refuses to answer	
Occupation	
State employee	83 (10.8)
Private sector worker	358 (46.6)
budget employee	51 (6.6)
Entrepreneur	102 (13.3)
Agricultural worker	4 (0.5)
Student	48 (6.2)
Homeowner	3 (0.4)
Pensioner	46 (6.0)
Unemployed (able to work)	62 (8.1)
Unemployed (unable to work)	11 (1.4)
Refuses to answer	1 (0.1)
Currently smoking any tobacco products	
Yes	224 (29.1)
No	545 (70.9)
Alcohol consumption in the last 30 days	
Yes	204 (26.5)
No	149 (19.4)
Do not consume	416 (54.1)
The presence of high‐intensity work in which breathing or heart rate increases significantly (e.g., heavy lifting, field, earthwork, or construction work) and which lasts continuously for at least 10 min	
Yes	147 (19.1)
No	622 (80.9)
Number of days in a typical week for high‐intensity physical labor at work	
1	9 (1.2)
2	16 (2.1)
3	16 (2.1)
4	11 (1.4)
5	34 (4.4)
6	23 (3.0)
7	41 (5.3)
Amount of time in a typical week spent sitting or reclining (hours)?	
0	11 (1.4)
1	37 (4.8)
2	87 (11.3)
3	79 (10.3)
4	100 (13.0)
5	101 (13.1)
6	99 (12.9)
7	38 (4.9)
8	91 (11.8)
9	32 (4.2)
10	46 (6.0)
11	5 (0.7)
12	29 (3.8)
14	5 (0.7)
15	4 (0.5)
16	1 (0.1)
20	1 (0.1)
23	1 (0.1)
77	1 (0.1)

In terms of educational attainment, the overwhelming majority in *n* = 339 (44.1%) had a higher education, and *n* = 180 (23.4%) of the respondents had a postgraduate education. And only completed secondary education (11 classes) was determined by *n* = 187 (24.3%) of the respondents.

By nationality, among all *n* = 769 respondents, in *n* = 611 (79.5%) cases, the respondents were Kazakhs, and *n* = 78 (10.1%) Russians. Uzbeks and Uighurs accounted for *n* = 21 (2.7%) and *n* = 19 (2.5%), respectively. Among the respondents, a small proportion were Tatars with an indicator of *n* = 6 (0.8%), and other nationalities were identified among the respondents in *n* = 26 (3.4%) cases.

According to marital status, *n* = 536 (69.7%) of the respondents were married, and in slightly more than ¼ in *n* = 194 (25.2%) cases, the respondents were single. *n* = 24 (3.1%) of the participants indicated that they were divorced, and *n* = 6 (0.8%) respondents indicated that they were married but separated.

By type of employment, the vast majority of the interviewed men in *n* = 358 (46.6%) were employees of the private sector, and *n* = 83 (10.8%) of the participants were civil servants. *n* = 102 (13.3%) of the respondents were entrepreneurs, and *n* = 51 (6.6%) were state employees. Students and pensioners accounted for *n* = 48 (6.2%) and *n* = 46 (6.0%), respectively. Among the respondents, the unemployed, able to work amounted to *n* = 62 (8.1%), and the unemployed, unable to work were equal to *n* = 11 (1.4%).

Smoking of any tobacco products was currently confirmed by *n* = 224 (29.1%) and denied by *n* = 545 (70.9%) of respondents.

Among the respondents, *n* = 416 (54.1%) respondents did not drink alcohol. While the number of participants who consumed alcohol in the last 30 days equal to *n* = 204 (23.4%) was higher compared to *n* = 149 (19.4%) nondrinking respondents.

In terms of physical activity, *n* = 147 (19.1%) respondents confirmed the presence of high‐intensity activity, in which breathing or heart rate increases significantly (e.g., weight lifting, field, earthwork, or construction work) and which lasts continuously for at least 10 min, and *n* = 622 (80.9%) denied.

Among respondents who confirmed high‐intensity physical activity, *n* = 41 (5.3%) respondents spent 7 days in a typical week doing high‐intensity physical activity at work, and *n* = 34 (4.4%) 5 days. *n* = 9 (1.2%) of the respondents spent only 1 day in a typical week doing high‐intensity physical labor at work.

In terms of the amount of time in a typical week spent sitting or reclining, *n* = 100 (13.0%) and *n* = 101 (13.1%) respondents spent an average of 4 and 5 h per week, respectively. While *n* = 99 (12.9%) of the respondents spent 6 h per week sitting or reclining, and *n* = 29 (3.8%) spent 12 h.

The results of checking the reliability of the questionnaire are presented in Table 2. The reliability of individual questions was almost the same. Cronbach's *α* was 0.791 for the total score.

**Table 2:** Reliability test.

Items	Cronbach's *α*	CI 95%
Sexual drive	0.786	0.724–0.879
Erections	0.798	0.654–0.897
Ejaculations	0.691	0.549–0.910
Problem assessment	0.724	0.659–0.891
Overall satisfaction	0.854	0.871–0.985
Total mean score	0.791	0.798–0.915

Abbreviation: CI, confidence interval.

The evaluation of the results of the survey according to the BSFI questionnaire on sexual dysfunction, depending on the city of residence, is shown in Table 3.

**Table 3:** Assessment of the results of the survey on the BSFI questionnaire on sexual dysfunction, depending on the city of residence.

Indicator	Almaty	Astana	Shymkent	Total
Sexual drive	2.23 ± 0.75	2.56 ± 0.68	2.41 ± 0.43	2.38 ± 0.12
Erections	2.98 ± 0.95	2.85 ± 0.85	2.56 ± 0.97	2.79 ± 0.57
Ejaculations	3.11 ± 0.98	3.09 ± 0.92	3.25 ± 0.52	3.03 ± 0.76
Problem assessment	2.89 ± 0.92	2.92 ± 0.66	2.96 ± 0.39	2.93 ± 0.88
Overall satisfaction	2.98 ± 0.91	3.01 ± 0.97	3.17 ± 0.48	3.08 ± 0.74
Total mean score	2.69 ± 0.87	2.69 ± 0.95	2.82 ± 0.92	2.73 ± 0.92

Abbreviation: BSFI, Brief Sexual Function Inventory.

In terms of sexual drive, the average overall score was 2.38 ± 0.12, among which this indicator for respondents from Astana is 2.56 ± 0.68 was higher in comparison with the scores of respondents from Almaty (2.23 ± 0.75) and Shymkent (2.41 ± 0.43). According to the following indicator, as erections among respondents from Shymkent (2.56 ± 0.97), this indicator was lower than the scores of respondents from the cities of Almaty (2.98 ± 0.95) and Astana (2.85 ± 0.85). While the scores for the ejaculations parameter were lower among respondents from Astana (3.09 ± 0.92), in contrast to the indicators of respondents from the cities of Almaty (3.11 ± 0.98) and Shymkent (3.25 ± 0.52). And the scores for the problem assessment were relatively lower for respondents from Almaty (2.89 ± 0.92) in comparison with the scores of respondents from the cities of Astana (2.92 ± 0.66) and Shymkent (2.92 ± 0.66). The scores for the indicator “Overall satisfaction” in comparison with the answers of respondents from Almaty (2.98 ± 0.91) were higher for respondents from Shymkent (3.17 ± 0.48).

Thus, the respondents from Shymkent had an average total score on the BSFI questionnaire of 2.82 ± 0.92 (*p* ≤ 0.05), which was higher than the total scores of respondents from the cities of Almaty (2.69 ± 0.87) and Astana (2.69 ± 0.95).

The results of a multivariate logistic analysis of factors in the development of sexual dysfunction in study participants who scored below ≤2 points in the survey are presented in Table 4.

**Table 4:** Multivariate logistic analysis by dysfunction, that is, those with an overall average score below 2 (≤2 points).

Indicator	Value		
	OR	95% CI	*p*
Age, years			
18–24	0.79	0.2–3.07	0.742
25–29	1.12	0.82–1.54	0.851
30–34	0.97	0.41–1.39	0.677
35–39	5.65	0.07–8.91	0.526
40–44	0.89	0.31–1.57	0.158
45–49	4.52	0.83–6.94	0.987
50–54	1.57	0.37–2.08	0.231
55–59	2.59	0.78–3.22	0.024*
60–64	1.28	0.01–0.94	0.036*
65 and older	1.81	0.57–1.24	0.001*
Body mass index			
<25	0.89	0.49–1.09	0.64
25–29.9	1.89	1.42–2.06	0.75
>30	1.84	0.58–1.21	0.01*
Smoking	1.42	0.79–1.97	0.001*
Alcohol consumption	1.37	0.32–3.21	0.12
Nationality			
Kazakhs	2.11	0.18–24.9	0.79
Russians	1.94	0.67–13.2	0.82
Other	0.81	0.43–5.23	0.63
Marital status			
Single	0.99	0.31–1.54	0.06
A family	1.513	1.09–1.93	0.47
Divorced/widower	1.258	0.85–1.49	0.82
Refuses to answer	0.137	0.07–0.24	0.39
Presence of high‐intensity activity	1.58	0.04–1.91	0.04*
Sitting/lying down more than 6 h a day	1.49	0.89–1.97	0.01*

Abbreviations: CI, confidence interval; OR, odds ratio.

* *p* < 0.05 was considered statistically significant.

By age categories, a statistically significant relationship was found between sexual dysfunction and age indicators over 55 years. Thus, the development of sexual dysfunction in the age group 55–59 years (odds ratio [OR]: 2.59; 95% CI: 0.2–3.7) was statistically significant (*p* = 0.023). Also, in the age groups 60–64 years (OR: 1.28; 95% CI: 0.01–0.94) and in participants aged 65 years and older (OR: 1.81; 95% CI: 0.57–1.24), statistically significant values for the development of sexual dysfunction were determined, with *p* = 0.036 and *p* = 0.001, respectively.

Based on BMI results, participants with overweight (BMI > 30) had a statistically significant relationship with sexual dysfunction with an OR of 1.84; 95% CI: 0.58–1.21 (*p* = 0.01).

According to the smoking factor, in study participants with sexual dysfunction who scored below ≤2 points in the survey, a statistically significant relationship was also determined OR 1.42; 95% CI: 0.79–1.97 (*p* = 0.001).

However, the presence of high‐intensity activity in study participants had a statistically significant relationship with the development of sexual dysfunction with OR values of 1.58; 95% CI: 0.04–1.91 (*p* = 0.04). In addition, the effect of physical inactivity (sitting/lying more than 6 h a day) was also associated with the presence of sexual dysfunction (OR: 1.49; 95% CI: 0.89–1.97), which was considered a statistically significant difference (*p* = 0.01).

## 4 DISCUSSION

To the best of our knowledge, this is the first study examining the sexual function of male participants using tools specifically designed to assess male sexual function in the three biggest cities of Kazakhstan.

According to the results acquired, it was revealed that the prevailing majority of participants (44.1%) had higher education. In a previously published study, it was indicated that men with a college education had a lower tendency to develop erectile dysfunction compared to those who did not have a high school diploma, but this decrease was not statistically significant. One explanation was that higher education is an indicator of higher socioeconomic status, which is associated with positive lifestyle factors and better access to health care.^16^

By nationality, among all *n* = 769 respondents, in *n* = 611 (79.5%) cases, the respondents were Kazakhs, and *n* = 78 (10.1%) Russians. By marital status, the vast majority of 69.7% of respondents were married.

As a risk factor, smoking any tobacco products was confirmed by 29.1% of respondents, while among the respondents 54.1% of respondents did not drink alcohol. According to the level of physical activity, 19.1% of respondents confirmed the presence of work with a high‐intensity load. In terms of the amount of time spent sitting or reclining in a typical week, approximately 13.0% of respondents spent an average of 4 and 5 h per week.

Since sexual dysfunction is a complex set of symptoms, doctors should diagnose the underlying pathologies, and risk factors that can lead to it, instead of focusing only on finding a cure.^17^

The use of the BSFI questionnaire was justified in view of the fact that Cronbach's *α* was 0.791 for the total score, indicating the applicability and validity of this questionnaire in this study. In our study, according to the results of the survey, in general, for all three cities, the average indicators of the sexual drive were 2.38 ± 0.12, erections 2.79 ± 0.57, ejaculations 3.03 ± 0.76, problem assessment 2.93 ± 0.88, and overall satisfaction 3.08 ± 0.74.

According to the results of the survey on the BSFI questionnaire, the respondents from Shymkent had an average total score on the BSFI questionnaire of 2.82 ± 0.92, (*p* ≤ 0.05), which was higher than the total scores of respondents from the cities of Almaty (2.69 ± 0. 87) and Astana (2.69 ± 0.95).

Sexual function is known to decline with age.^18^ The findings indicated that among study participants who scored below ≤2 points according to the BSFI survey, a statistically significant relationship was found between sexual dysfunction and age indicators over 55 years.

According to some reports, the frequency of sexual dysfunction increases not only with age, but can also be associated with a number of diseases, such as diabetes, tobacco abuse, metabolic syndrome, cardiovascular disease, and obesity.^19^

We observed a direct statistically significant relationship between overweight (BMI > 30) and sexual dysfunction (*p* = 0.01). These data are consistent with the results of previous studies, where it was assumed that an obese man has the same degree of sexual dysfunction as a nonobese man, about 20 years older in age.^13^ In addition, in the Massachusetts Male Aging Study, the overall prevalence of erectile dysfunction was 17%, but it increased to 45% in subjects with BMI values of 30 kg/m^2^.^20^ Despite the fact that the reason for the influence of excess weight on the manifestation of sexual dysfunction in men is still not completely clear, there are some suggestions of a connection with a decrease in the concentration of free and total testosterone in the blood serum and a decrease in sex hormone‐binding globulin in obese men. Moreover, the rate of estrogen production can also elevate with increasing obesity, possibly due to the activation of androgens by adipocytes.^21^

Smoking, as a possible risk factor, also showed a statistically significant association with sexual dysfunction in our study participants who scored below ≤2 on the survey (OR: 1.42; 95% CI: 0.79–1.97). This is also consistent with the results of a previously published meta‐analysis of more than 28,000 participants, which found that compared with nonsmokers, the OR risk of developing erectile dysfunction in cohort prospective studies was 1.51 (95% CI: 1.34–1.71) for smokers and 1.29 (95% CI: 1.07–1.47) for ex‐smokers.^22^ In addition, one study examined the relationship between cumulative exposure (pack‐years based on reported smoking duration and intensity) and erectile dysfunction in 2115 randomly selected Minnesota White men aged 40–79. The ORs for erectile dysfunction in men who smoked 1–12.5 pack‐years, 12.6–29.0 pack‐years, and 29 pack‐years after adjusting for age and comorbidities were 0.96, 1.44, and 1.60, respectively.^23^ According to some reports, this can be associated with the effect of nicotine on increasing the tone of the sympathetic nervous system, which causes vasoconstriction and thereby reduces blood flow to the penis, and nicotine contributes to endothelial dysfunction, which worsens erectile function.^16^

The results of some national studies showed the relationship between an increase in the chances of developing sexual dysfunction in men, depending on race/ethnicity.^24^ However, in our study, according to such parameters as nationality, marital status, a causal relationship with sexual dysfunction was not determined (*p* > 0.05).

The presence of high‐intensity activity in the study participants had a statistically significant relationship with the development of sexual dysfunction (*p* = 0.04).

Hypodynamia also had a relationship with the presence of sexual dysfunction, which was regarded as a statistically significant difference (*p* = 0.01). Literature review shows that sexual dysfunction in middle‐aged men is often an early manifestation of endothelial damage, and physical activity can improve both erectile and endothelial dysfunction.^17^ In a previously published study, it was noted that intense and moderate physical activity is associated with a lower risk of developing erectile dysfunction. It increases endothelial NO production and reduces oxidative stress and levels of inflammatory markers, including proinflammatory cytokines and C‐reactive protein.^25^ The results of a prospective, randomized study in Italy in moderately obese sedentary men demonstrated the role of lifestyle changes (weight loss, physical activity) in reversing erectile dysfunction.

Since sexual problems have a direct and indirect impact on various aspects of life, it is necessary to educate people to identify, diagnose, treat, and prevent such dysfunctions. The vast majority of risk factors for sexual dysfunction in men, such as smoking, physical inactivity, and obesity, are modifiable.^25^ Therefore, it is necessary to pay attention to preventive measures to eliminate these risk factors.

## 5 CONCLUSIONS

We were able to identify the indicators that are important for the prevention of sexual dysfunction in men. The various risk factors were evaluated using an adjusted BSFI questionnaire. According to our findings, males who are over 50, smoke, are overweight, work at high intensities, are inactive, and are also at risk for sexual dysfunction.

Early initiation into a healthy lifestyle may be the best approach to reduce the burden of sexual dysfunction on the health and well‐being of men over 50 years of age. Further ongoing community programs are needed to highlight the importance of addressing sexual dysfunction, decreasing smoking, and educating on the importance of physical activity.

### 5.1 Study limitations

This study has a few limitations. Due to the inclusion of respondents from only three cities in this study, the sample size of participants was insufficient. In addition, some laboratory parameters, such as testosterone levels and other hormones, were not covered by this study. Aside from that, due to the survey methodology, the presence of the level of sexual dysfunction of the study participants was based only on the answers of the respondents, without verification of this pathology by a urologist.

## AUTHOR CONTRIBUTIONS

**Marat Sikhymbaev**: Data curation; resources; software; validation; writing—original draft; writing—review and editing. **Dinara Ospanova**: Resources; software; supervision; writing—original draft. **Andrey Grzhibovsky**: Methodology; validation; visualization; writing—original draft. **Merkhat Akkaliyev**: Investigation; validation; visualization; writing—original draft. **Turar Kurmanbekov**: Methodology; software; writing—original draft; writing—review and editing. **Shynar Tanabayeva**: Methodology; writing—original draft; writing—review and editing. **Timur Saliev**: Funding acquisition; resources; software; validation; writing—review and editing. **Sagat Altynbekov**: Formal analysis; investigation; validation; writing—review and editing. **Ildar Fakhradiyev**: Methodology; validation; writing—original draft.

## CONFLICT OF INTEREST STATEMENT

The authors declare no conflict of interest.

## ETHICS STATEMENT

The study was approved by the Local Ethics Committee of S.D. Asfendiyarov Kazakh National Medical University, Almaty, Republic of Kazakhstan (protocol of the Local Ethics Commission No. 12 (118) of 28.09.2021). In addition, the study was registered with ClinicalTrials.gov (NCT05122832).

## TRANSPARENCY STATEMENT

The lead author Ildar Fakhradiyev affirms that this manuscript is an honest, accurate, and transparent account of the study being reported; that no important aspects of the study have been omitted; and that any discrepancies from the study as planned (and, if relevant, registered) have been explained.

## Data Availability

All the available data were indicated within the manuscript text.

## APPENDIX A

See Table A1.

**Table A1:** Brief Sexual Function Inventory questionnaire.

*Sexual drive*				
1 During the past 30 days, how many days have you felt sexual drive?				
No days	Only a few days	Some days	Most days	Almost everyday
0	1	2	3	4
2 During the past 30 days, how would you rate your level of sexual drive?				
Not at all	Low	Medium	Medium high	High
0	1	2	3	4
*Erections*				
3 Over the past 30 days, how often have you had partial or full sexual erections when you were sexually stimulated in any way?				
Not at all	A few times	Fairly often	Usually	Always
0	1	2	3	4
4 Over the past 30 days, how often have you had erections; how often were they firm enough to have sexual intercourse?				
Not at all	A few times	Fairly often	Usually	Always
0	1	2	3	4
5 How much difficulty did you have getting an erection during the last 30 days?				
No erections at all	A lot	Some	Little	No difficulty
0	1	2	3	4
*Ejaculation*				
6 Over the past 30 days how much difficulty have you had in ejaculating when you have been sexually stimulated?				
Have not had sexual stimulation	A lot	Some	Little	No difficulty
0	1	2	3	4
7 In the past 30 days, how much did you consider the amount of semen you ejaculate?				
Did not climax	Big problem	Medium problem	Small problem	No problem
0	1	2	3	4
*Problem assessment*				
8 How big a problem has it been for you to decrease your sex drive over the past 30 days?				
Big problem	Medium problem	Small problem	Very small problem	No problem
0	1	2	3	4
9 In the past 30 days, to what extent have you considered your ability to get and keep an erection a problem?				
Big problem	Medium problem	Small problem	Very small problem	No problem
0	1	2	3	4
10 In the past 30 days, to what extent have you considered your ejaculation to be a problem?				
Big problem	Medium problem	Small problem	Very small problem	No problem
0	1	2	3	4
*Overall satisfaction*				
11 Overall during the past 30 days, how satisfied have you been with your sex life?				
Absolutely not satisfied	Mostly not satisfied	Mixed feeling	Mostly satisfied	Completely satisfied
0	1	2	3	4

## APPENDIX B

See Table B1.

**Table B1:** Brief Sexual Function Inventory questionnaire in the Kazakh language.

*Жыныстық құмарлық*				
1 Соңғы 30 күн ішінде Сіз жыныстық құмарлықты қаншалықты жиі сезіндіңіз?				
Бір рет те емес	Бір немесе екі рет	Бірнеше рет	Көп рет	Күн сайын дерлік
0	1	2	3	4
2 Соңғы 30 күн ішінде Сіздің жыныстық құмарлығыныздың күшін қалай бағалауға болады?				
Мүлем жоқ	Төмен	Орташа	Орташадан жоғары	Жоғары
0	1	2	3	4
*Эрекциялар*				
3 Соңғы 30 күн ішінде жыныстық ынталандыруға ұшыраған кезде Сізде жартылай немесе толық эрекция қаншалықты жиі болды?				
Бір рет те емес	Бірнеше рет	Өте жиі	Шамамен әрдайым дерлік	Әрдайым
0	1	2	3	4
4 Соңғы 30 күн ішінде Сізде эрекциялар болған кезде, олар жыныстық қатынасқа түсу үшін қаншалықты күшті болды?				
Бір рет те емес	Бірнеше рет	Өте жиі	Шамамен әрдайым дерлік	Әрдайым
0	1	2	3	4
5 Соңғы 30 күн ішінде Сізге эрекцияға жету қаншалықты қиын болды?				
Эрекция мүлдем болмады	Үлкен қиындықтар	Кейбір қиындықтар	Кішкене қиындықтар	Ешқандай проблема болған жоқ
0	1	2	3	4
*Эякуляция*				
6 Соңғы 30 күн ішінде жыныстық ынталандыру кезінде Сізге эякуляция жасау қаншалықты қиын болды?				
Жыныстық ынталандыру мүлдем болмады	Үлкен қиындықтар	Кейбір қиындықтар	Кішкене қиындықтар	Ешқандай проблема болған жоқ
0	1	2	3	4
7 Соңғы 30 күн ішінде эякуляция кезінде бөлінетін сперманың мөлшері Сізді алаңдатты ма?				
Эякуляция мүлдем болмады	Қатты мазалады	Орташа алаңдаушылық тудырды	Аздап алаңдаушылық тудырды	Мазаламады
0	1	2	3	4
*Проблеманы бағалау*				
8 Соңғы 30 күн ішінде жыныстық құмарлықтың төмендеуі Сіз үшін қаншалықты үлкен проблема болды?				
Үлкен проблема	Орташа маңызды проблема	Кішкентай проблема	Өте аз проблема	Проблема болған жоқ
0	1	2	3	4
9 Соңғы 30 күнде Сіз өзіңіздің эрекцияға жету және оны сақтау қабілетіңізді қаншалықты проблема деп санадыңыз?				
Үлкен проблема	Орташа маңызды проблема	Кішкентай проблема	Өте аз проблема	Проблема болған жоқ
0	1	2	3	4
10 Соңғы 30 күнде Эякуляция сапасы Сіз үшін қаншалықты үлкен проблема болды?				
Үлкен проблема	Орташа маңызды проблема	Кішкентай проблема	Өте аз проблема	Проблема болған жоқ
0	1	2	3	4
*Жалпы қанағаттану*				
11 Жалпы, соңғы 30 күнде Сіз жыныстық өміріңізге қаншалықты қанағаттандыңыз?				
Мүлдем қанағаттанбадым	Көп бөлігінде қанағаттанбадым	Аралас сезім тудырады	Көп бөлігінде қанағаттандым	Толықтай қанағаттандым
0	1	2	3	4
